# Laser–material interactions in liquids for the synthesis of nanomaterials: current status and perspectives

**DOI:** 10.3762/bjnano.17.38

**Published:** 2026-05-04

**Authors:** Carlos Doñate Buendia, Bilal Gökce, Leonid V Zhigilei

**Affiliations:** 1 GROC·UJI, Institute of New Imaging Technologies, University Jaume I, Av. de Vicent Sos Baynat, s/n, 12071 Castellón, Spain; 2 Chair of Materials Science and Additive Manufacturing, School of Mechanical Engineering and Safety Engineering, University of Wuppertal, 42119 Wuppertal, Germany; 3 Department of Materials Science and Engineering, University of Virginia, 395 McCormick Road, Charlottesville, Virginia 22904-4745, USA

**Keywords:** colloidal nanoparticles, green synthesis of nanoparticles, laser ablation in liquids, laser–matter interactions, laser processing in liquids

In the ever-evolving landscape of materials science and nanotechnology, laser synthesis and processing of colloids (LSPC) has emerged [1] as a powerful and versatile technique for producing high-purity, surfactant-free nanoparticles from a wide range of materials, including metals [2–3], oxides [4–5], semiconductors [6–7], and organics [8–9]. In addition, LSPC enables the synthesis of multicomponent nanomaterials, such as binary [10–11], ternary [12–13], and compositionally complex alloys [14–15], with a high degree of control over their composition.

The field of LSPC encompasses several techniques with distinct target configurations and laser irradiation regimes. Irradiation of a solid target submerged in a liquid with intense laser pulses is referred to as laser ablation in liquids (LAL) [16], an established technique for the efficient generation of surfactant-free, high-purity colloidal nanoparticles. Laser fragmentation in liquids (LFL), in turn, involves the irradiation of colloidal micro- or nanoparticle suspensions with high-intensity pulses above the fragmentation threshold, leading to size reduction through photomechanical or photothermal mechanisms [17]. Laser melting in liquids (LML) employs lower pulse intensities to increase particle size [18] and/or modify crystallinity and morphology through melting and resolidification [19]. At moderate laser fluences, close to or even below the melting threshold, irradiation can induce changes in atomic structure, enabling defect engineering [20].

The broad materials library accessible through LSPC, combined with the wide range of laser, target, and solvent parameters, creates a complex multidimensional optimization space governing nanoparticle size, internal structure, composition, and synthesis productivity [21]. This complexity has driven major efforts within the LSPC community toward elucidating the roles of the solvent environment [6,22], target geometry [23], and irradiation conditions [24] in nanoparticle formation. Advancing these efforts requires a fundamental understanding of the underlying processes, motivating computational and experimental studies focused on the mechanisms governing nanoparticle synthesis.

Theoretical and computational studies of LSPC have provided important insights into the emergence of cavitation bubble and nanoparticle formation mechanisms in LAL [25–28], nanoparticle fragmentation intertwined with the generation and collapse of nanobubbles in LFL [29], as well as defect generation during laser-induced melting and resolidification in liquids [30–31]. Experimental investigation of these processes, however, requires characterization techniques capable of probing the initial stages of nanoparticle synthesis with high temporal and/or spatial resolution [32–33]. In particular, accessing the picosecond time window needed to capture the initial interaction of the ablation plume with the liquid environment, which defines the subsequent longer-term cavitation bubble dynamics, remains challenging even for shadowgraphy employing ultrafast cameras [34]. Pump–probe reflectometry provides the required picosecond and even femtosecond resolution [35]. However, interpretation of the multi-stage reflectivity response is nontrivial and often requires input from computational modeling [33,36]. At longer timescales, shadowgraphy offers valuable information on bubble evolution [37], although processes occurring within the bubble remain largely inaccessible and necessitate the use of X-ray probing to elucidate nanoparticle growth mechanisms [38].

The complexity of LSPC processes and the wide parameter space call for close integration of computational and experimental efforts, where coordinated design of real and in silico experiments can significantly enhance opportunities for model validation and reliable interpretation of experimental data [33]. Representative examples of this approach include the combination of pump–probe measurements with atomistic simulations to reveal spallation and phase explosion regimes during pulsed laser ablation of Fe–Ni alloys [39], the identification and experimental confirmation of two mechanisms of nanoparticle generation in picosecond laser ablation in liquids [40], the elucidation of processes responsible for the formation of periodic surface structures on Cr targets irradiated by femtosecond pulses in water [41], and the integration of X-ray probing with simulations to study the transition from melting to explosive fragmentation of Au nanoparticles under picosecond laser irradiation in water [42]. Further advances in LSPC can be facilitated by data-driven machine learning approaches, which provide new pathways for optimizing synthesis parameters toward targeted size, composition, phase, and productivity, as demonstrated, for example, in tuning the oxidation state of Cu nanoparticles generated by LAL [43].

Despite significant progress in understanding LSPC mechanisms, key challenges remain in controlling nanoparticle functionality. Although LSPC is valued for producing high-purity materials, functional applications often require incorporation of additives for size control [44], enhanced colloidal stability [45], or specific surface functionalization [46]. For example, the use of scavengers to capture reactive species can influence nanoparticle size [47] and increase productivity [48]. Alternatively, employing organic solvents instead of water provides opportunities to produce carbon-encapsulated core−shell nanostructures [49] and enables control over oxidation [50], surface chemistry [51], and transferability between solvents [52].

The versatility of LSPC in terms of materials, solvents, and processing parameters has enabled a wide range of applications [53]. These include catalysis [54] (e.g., for oxygen [55] and hydrogen evolution reactions [56] in hydrogen production), sensing (e.g., surface-enhanced Raman spectroscopy [51] for detection of pollutants and optical sensing of glucose [57]), generation of soft magnets for magnetocaloric applications [58], fabrication of photodetectors [59], nanoscale agents for photodynamic [60] and neutron capture therapy [61], incorporation of nanoparticles into solar cells [62], light-harvesting nanofluids [63], and materials with enhanced mechanical [64] or antibacterial [65] properties produced by additive manufacturing.

A major bottleneck for the broader adoption of LSPC remains productivity. In contrast to chemical synthesis routes, nanoparticle yields in LAL have traditionally been limited to the mg/h range. Advances in high-repetition-rate laser systems, fast beam scanning, and flow-cell designs have enabled scaling to g/h production rates [66–67]. Even a higher 10 g/h productivity has recently been demonstrated in microparticle LFL using high pulse energy (>20 mJ) nanosecond laser systems [68]. While these advances represent an important step toward industrial relevance, the cost of high-power and high-repetition-rate laser systems remains a significant barrier, despite the ongoing decrease in their price.

Emerging strategies aim to increase productivity while reducing system complexity and cost. One promising approach is multiple-beam LAL (MB-LAL) using diffractive optical elements [69]. The static diffractive optical elements have at least an order of magnitude lower cost than the fast-scanning systems and have an inherent benefit: the possibility to use lower repetition rates while employing the optimum processing fluence per beam [70]. More broadly, beam shaping techniques are gaining attention as a means to improve process control and efficiency [24]. For example, non-Gaussian beam profiles, such as donut-shaped beams, have been shown to modify the nanoparticle size distribution [71]. The balance between implementation complexity, cost, productivity, and the degree of control over nanoparticle size, shape, and phase will ultimately determine the most effective strategies.

Looking forward, LSPC holds strong promise as a sustainable and environmentally friendly approach for nanomaterial production, eliminating the need for hazardous precursors and post-processing steps. To fully realize this potential, the field must transition from laboratory-scale demonstrations to scalable industrial processes. Achieving this goal will require continued advances in fundamental understanding, integration of computational and experimental tools, and development of cost-effective, high-throughput technologies. Laser processing in liquids is not merely a synthesis technique – it is an enabling platform for innovation in the development of next-generation nanomaterials, and its full potential is only beginning to be realized.

Carlos Doñate Buendia, Bilal Gökce and Leonid V. Zhigilei

Castellón, Wuppertal and Charlottesville, March 2026

## Data Availability

Data sharing is not applicable as no new data was generated or analyzed in this study.
